# Neoadjuvant Chemoradiotherapy With Simultaneous Integrated Boost in Locally Advanced Cervical Cancer: Long Term Results of a Single-Center Experience

**DOI:** 10.3389/fonc.2022.883965

**Published:** 2022-05-05

**Authors:** Alessia Nardangeli, Rosa Autorino, Luca Boldrini, Maura Campitelli, Sara Reina, Gabriella Ferrandina, Nicolò Bizzarri, Luca Tagliaferri, Gabriella Macchia, Vincenzo Valentini, Maria Antonietta Gambacorta

**Affiliations:** ^1^UOC Radioterapia Oncologica, Dipartimento Diagnostica per Immagini, Radioterapia Oncologica ed Ematologia, Fondazione Policlinico Universitario Agostino Gemelli Istituto di Ricovero e Cura a Carattere Scientifico (IRCCS), Roma, Italy; ^2^Dipartimento Universitario di Scienze Radiologiche ed Ematologiche, Università Cattolica del Sacro Cuore, Roma, Italy; ^3^UOC Ginecologia Oncologica, Dipartimento per la Salute della Donna e del Bambino e della Salute Pubblica, Fondazione Policlinico Universitario A. Gemelli, Istituto di Ricovero e Cura a Carattere Scientifico (IRCCS), Roma, Italy; ^4^Radiation Oncology Unit, Gemelli Molise Hospital, Università Cattolica del Sacro Cuore, Campobasso, Italy

**Keywords:** cervical cancer, neoadjuvant chemoradiation, simultaneous integrated boost, SIB-VMAT, volumetric modulated arc therapy

## Abstract

Aim of this study was to analyze the efficacy and tolerability of simultaneous integrated boost volumetric modulated arc therapy (SIB-VMAT) associated with cisplatin-based chemotherapy in preoperative setting of patients with locally advanced cervical cancer (LACC). From June 2013 to September 2019, we analyzed patients with LACC who had undergone neoadjuvant chemoradiation (CRT). A radiation dose of 39.6 Gy, 1.8 Gy/fraction was delivered to the pelvis plus a radiation dose to the primary tumor delivered with SIB-VMAT strategy for a total of 50.6Gy, 2.3Gy/fraction in 25 fractions. Cisplatin-based chemotherapy was delivered combined with radiotherapy. Radical hysterectomy plus pelvic with or without aortic lymphadenectomy was performed within 7 to 8 weeks from CRT. One hundred forty-eight patients (median age: 49.5 years; FIGO stage IB2: 7, IIA: 8, IIB: 106, IIIA: 5; IIIB: 16; IVA: 5, IVB: 1; N0: 56, N1: 92) were analyzed. The treatment was well tolerated with good compliance: no grade 3/4 gastrointestinal or genitourinary toxicity was reported; grade 3 neutropenia was described in five cases. Pathological complete response (pCR) was documented in 68 cases (46%) and 32 patients (21.6%) had microscopic residual disease. Pathological nodal involvement was observed in 23 patients (15.5%). At median follow-up of 59 months (range: 27-100), the 3-year local control was 78.5%, whereas the 3-year metastasis-free survival was 70.5%. The 3-year overall survival rate was 89.0%. Neoadjuvant CRT with SIB-VMAT followed by radical surgery results in a high rate of pathologically assessed complete response and a very encouraging local control rate, with acceptable toxicity.

## Introduction

Cervical cancer (CC) represents the fourth leading cause of cancer death in women, with 311,000 deaths in 2018 worldwide (1). Treatment depends mainly on the stage of the tumor at diagnosis, as assessed by the International Federation of Gynecology and Obstetrics (FIGO) 2009 staging system (2). Definitive chemoradiotherapy (CRT) has represented the gold standard of treatment in locally advanced cervical cancer since 1999, with a gain in overall survival (OS) and disease-free survival (DFS) compared to radiotherapy alone. Despite these results, the 5-year overall survival still remains around 70% in this subgroup of patients (3).

Several studies were conducted using neoadjuvant chemotherapy or concurrent chemoradiotherapy followed by radical surgery in order to try to improve outcomes of patients with locally advanced cervical cancer (4–10). The GYNECO 02 trial demonstrated no therapeutic impact of radical surgery following CRT in patients with clinical and radiological complete response after neoadjuvant treatment (8). Cetina et al. compared the role of brachytherapy and radical surgery after CRT, showing no differences in outcomes between the two therapeutic options (9). Indeed, radical surgery after neoadjuvant treatment could be useful removing chemo- and radio-resistant tumor foci, obtaining pathological response to the pre-operative therapy, and determining pathological assessment in the view of a more tailored patient treatment (11, 12).

Several technical innovations have been introduced in the clinical routine of the radiation oncologist (13). Intensity-modulated radiotherapy (IMRT), such as volumetric arc radiotherapy or helical tomotherapy, allows an increase of the dose to the target volume and a reduction of the dose to the organs at risk (OARs), with less acute and late toxicity (14, 15). Therefore, the use of on-board cone-beam or fan-beam CT (image-guided RT, IGRT) in daily practice allows for a daily check of the patient’s setup and a reduction of the margins and consequently of the treatment volumes. Furthermore, conventional IMRT techniques allow the simultaneous delivery of different doses to different target volumes within a single fraction (Simultaneous Integrated Boost - SIB).

The phase I and II LARA-CC-1 trial was set up to investigate a regimen based on gross tumor volume (GTV)-accelerated fractionation and lymph node extended-field (LNEF) irradiation followed by radical surgery. The total dose of 45Gy (2.25Gy/fraction) to macroscopic tumor and 40Gy (2Gy/fraction) to lymph node station was established as the recommended dose, with a complete pathological response to treatment rate of 38.6% (16, 17). In this setting, a subsequent dose escalation study demonstrated that the SIB-IMRT technique is feasible and safe, but it’s unable to safely escalate dose beyond levels already achieved with three-dimensional conformal radiotherapy technique (18).

In this scenario, we retrospectively analyze the efficacy and tolerability of simultaneous integrated boost volumetric modulated arc therapy (SIB-VMAT) associated to cisplatin-based chemotherapy in our preoperative setting of patients with locally advanced cervical cancer.

## Materials and Methods

### Patients’ and Tumor Characteristics

Patients (pts) affected by locally advanced cervical carcinoma, with 2009 FIGO Stage from IB2 to IVB, treated in our Institution from June 2013 to September 2019 with neoadjuvant SIB-VMAT cisplatin-based CRT, were retrospectively analyzed. Inclusion criteria were histologically confirmed invasive carcinoma of the cervix, FIGO 2009 stage IB2-IV, and age ≥18 years. Exclusion criteria were the presence of distant metastasis (M1) and treatment with palliative intent.

### Medical Treatments

All patients underwent CT-based planning without intravenous contrast media in a supine position. Patients were asked to drink 500mL of water 30’ before CT planning and before each treatment session in order to obtain a reproducible full bladder. Furthermore, to limit inter-fraction and intra-fraction variability, an empty rectum was required. According to the consensus guidelines for the delineation of Clinical Target Volume (CTV) for intensity-modulated pelvic radiotherapy in the treatment of cervical cancer, CTVs were defined as follow: CTV1 includes primary tumor, CTV2 includes whole pelvis. In case of common iliac or lombo-aortic lymph nodes positivity at the staging imaging, lombo-aortic lymph nodes were added to the CTV2. Isotropic CTV to Planning Target Volume (PTV) margins of 5mm were adopted.

All PTVs were encompassed by a minimum of 95% of the prescribed dose. No more than 5% of any PTV received > 105% of its prescribed dose. Organ at risk, including rectum, bladder, femoral heads, and peritoneal bag were also contoured. The peritoneal bag was contoured from the axial slices situated 2cm above the most superior slice containing the CTV and continued to its most inferior extent in the pelvis. Rectum was contoured as a solid continuous structure and was defined from the sigmoid flexure to the anus. The bladder was also contoured as a solid continuous structure.

Patients underwent SIB-VMAT with a prescribing dose of 50.6 Gy at 2.3 Gy/fraction to the CTV1 and 39.6 Gy at 1.8 Gy/fraction to the CTV2 in 22 fractions.

Plans consisted of two arcs using 10-15-MV photon beams.

For quality assurance through treatment planning and delivery, two independent checks were performed by physicians and medical staff; daily setup reproducibility was checked.

Concomitant chemotherapy was delivered based on histology as follow:

- Weekly cisplatin at the dose of 40mg/m2 in patients with squamous cell carcinoma.
- Cisplatin (20 mg/m2, 2-h intravenous infusion) and 5-fluorouracil (1000 mg/m2, 24-h continuous intravenous infusion) both on first and last week of treatment for 4 consecutive days in patients with adenocarcinoma.

Neoadjuvant chemotherapy before CRT and surgery was administered in 37 (25%) patients with locally advanced disease for 6 consecutive weeks with paclitaxel (80mg/mq, 1-hour-infusion) and carboplatin (AUC2, 1-hour infusion).

### Surgery

Restaging was performed 5 to 6 weeks after completion of CRT with MRI and PET-CT. Patients achieving response or stable disease after treatment were triaged to radical hysterectomy according to Querleu and Morrow plus pelvic with or without aortic lymphadenectomy within 7 to 8 weeks after the completion of CRT (19).

Pathological response to treatment was evaluated analyzing uterus, vaginal cuff, parametria, and pelvic and aortic lymph nodes. Residual disease at any site was expressed in millimeters and response was defined as follow: complete, absence of any residual tumor after treatment at any site level (pCR); microscopical, persistent tumor foci of ≤3mm maximum dimension microscopic pathological response (PR1); macroscopic, persistent tumor foci of >3mm maximum dimension (pR2).

### Toxicity

Acute toxicity during CRT was assessed according to the RTOG criteria.

### Follow up

Follow-up during the first year after treatment completion was at 3-month intervals, gradually increasing over subsequent 6-month intervals. Disease status was determined clinically at each follow up. Complete response to treatment was confirmed by pathological analysis. Suspected local/regional and/or distant recurrence were confirmed with imaging (Magnetic Resonance Imaging, Positron Emission Tomography, Computed Tomography).

### Statistical Methods

Primary endpoint of this analysis was local control (LC). Secondary endpoints were overall survival (OS) and metastasis-free survival (MFS). LC was calculated from the date of surgery to the date of inside field relapse/progression of disease, or the date last seen. MFS was calculated from the date of surgery to the date of distant relapse or the date of the last follow-up. OS was calculated from the date of diagnosis to the date of death or the date of the last follow-up.

Median values and survival tables were computed using the product limit estimate by Kaplan–Meier method (20). Cox proportional hazards regression model was used to analyze the effect of covariate survival.

Statistical analysis was carried out using MedCalc Statistical Software version 15.8 (MedCalc Software bvba, Ostend, Belgium; https://www.medcalc.org; 2015).

## Results

We retrospectively analyzed the data of 148 patients affected by histologically proven cervical carcinoma with IB2-IVB FIGO 2009 stage. Median age was 49.5 years (range 20-78). One hundred six patients (71.6%) were FIGO Stage IIB, and 62.2% had radiographically positive lymph nodes at staging imaging. We included in our analysis one patient with IVB FIGO stage because of pelvic peritoneal metastasis which were encompassed by the clinical target volume. The majority of tumors were squamous carcinoma (N=128, 86.5%). Patients’ baseline characteristics and treatment details are summarized in **Table 1**.

**Table 1:** Patients’ baseline characteristics and treatment details.

All cases	148 pts
**Median Age** (Range)	49.5 (20-78)
**Histology**	
Squamous cell carcinoma	128 (86.5%)
Adenocarcinoma	17 (11.5%)
Other	3 (2.0%)
**FIGO^1^ Stage 2009**	
IB2	7 (4.7%)
IIA	8 (5.4%)
IIB	106 (71.6%)
IIIA	5 (3.4%)
IIIB	16 (10.8%)
IVA	5 (3.4%)
IV B	1 (0.7%)
**Nodal status at imaging**	
N0	56 (37.8%)
N1	92 (62.2%)
**Concomitant CT**^2^	
Weekly Cisplatin	131 (88.5%)
Cisplatin + 5fluorouracil	17 (11.5%)
**Median Days of interruption** (Range)	0 (0-14)
**Neoadjuvant CT**^2^	
Yes	37 (25%)
No	111 (75%)

^1^FIGO, International Federation of Gynecology and Obstetrics (Fédération Internationale de Gynécologie et d’Obstétrique); ^2^CT, chemotherapy.

Chemoradiation was well tolerated with good compliance. As described in **Table 2**, no grade 3 or 4 gastrointestinal or genitourinary toxicity were reported. Five patients developed a Grade 3 neutropenia which led to a temporary treatment interruption of a median of 8.5 days (range 0-10).

**Table 2:** Acute Toxicities during chemoradiotherapy.

Acute Toxicity	Grade	N° (%)
**Gastro-intestinal Toxicity**		
	G0	93 (62.8%)
	G1	45 (30.4%)
	G2	10 (6.8%)
	G3	0
	G4	0
**Genitourinary Toxicity**		
	G0	133 (89.9%)
	G1	12 (8.1%)
	G2	3 (2.0%)
	G3	0
	G4	0
**Haematological Toxicity**		
	G0	106 (71.6%)
	G1	28 (18.9%)
	G2	9 (6.1%)
	G3	5 (3.4%)
	G4	0

After imaging restaging, all pts successfully underwent radical surgery.

Pathological response was assessed in all patients receiving surgery: 68 patients (46%) showed pathological complete response (pCR) and 32 patients (21.6%) had a microscopic residual disease. Pathological nodal involvement was observed in 23 patients (15.5%) divided as follow: four patients of the pCR group, four patients of the pR1 group, and 15 patients of the pR2 group. (**Table 3**)

**Table 3:** Pathological response to treatment.

All cases	148 pts
**Group**	
pCR^1^	68 (46%)
pR1^2^	32 (21.6%)
pR2^3^	48 (32.4%)
**Pathological Nodal Status**	
N0	125 (84.5%)
N1	23 (15.5%)

^1^pCR: pathological complete response;^2^ pR1: microscopic residual disease; ^3^pR2: macroscopic residual disease.

According to the pathological response to treatment, adjuvant chemotherapy was administered in 40 patients (27%) with pR1-2 and/or nodal involvement, while five patients (3.4%) with pR2N0 underwent endovaginal brachytherapy.

As of July 2021, the median follow-up period was 59 months (range 27-100 months) in the overall series.

At the time of the analysis, the 1-year and 3-year local control (LC) were 88.5% and 78.5%, respectively (median LC: not reached), while the 1-year and 3-year metastasis-free survival (MFS) were 82.9% and 74.5%, respectively (median MFS: not reached). Death due to disease was recorded in 13 patients, with 3-year overall survival (OS) rates of 88.9% (median OS: not reached).

Patients with pathological nodal involvement experienced a statistically significant worse LC, OS, and MFS rate compared to those without positive lymph nodes after neoadjuvant CRT (P <.0001).

Concerning long-term toxicity, 13 patients (8.8%) developed grade 2 genitourinary toxicity, while no late gastro-intestinal or haematological toxicity were documented (data not shown).

## Discussion

Surgery represents the standard treatment for early stage cervical carcinoma, while exclusive chemoradiation followed by brachytherapy is considered the gold standard for locally advanced cervical cancer (3, 21–25). Despite the benefit on overall survival due to radical CRT, local relapse and distant metastasis represented the main cause of failure in LACC treatment (26). Furthermore nodal involvement represents one of the main factors influencing survival (27–29).

Several studies concerning radical surgery after CRT were conducted with the aim of evaluating the efficacy and safety of this kind of treatment and its impact on improving local control. As demonstrated by Classe et al., surgery allows evaluation of the pathological response to therapy and improves local control in the case of partial pathological response (6). This finding is closely associated with the clinical trend and allows for the evaluation of personalized prognostic profiles that lead to the definition of specific surveillance procedures or further treatments. A 2018 Meta-analysis showed that radical surgery following CRT seemed to reduce the risk of local recurrence compared to radical CRT followed by brachytherapy (30). Probably the data are due to the eradicating role that surgery could have on the residual cervical cancer or on the residual lymph node involvement after CRT.

Ferrandina et al. showed that the addition of concomitant boosts in accelerated fractionation modality to whole pelvis CRT followed by radical surgery results in a high rate of pathologically assessed complete response (50.5%) and a very encouraging local control rate, with an acceptable rate and profile of toxicity (31). The high tolerability and efficacy of this accelerated regimen was also confirmed by Macchia et al. in 2012, with a total of 59.1% complete/microscopic response to treatment (17).

In our series, pathological complete response rate combining both pCR and pR1 was 67.6%. These encouraging results could be related to the technological improvement that has led to the use of an accelerated regimen which allowed us to an intensification of the total dose at site of macroscopic disease through the use of SIB-VMAT and a reduction of treatment time with an increase in the effectiveness. This hypothesis is also supported by a previous study in which 45 Gy as a concomitant boost CRT delivered by a 3D technique did not seem sufficient to increase pCR rate (17).

As far as the primary and secondary endpoints are concerned, we reported 3-year local control, metastasis-free, and overall survival of 78.5%, 75%, and 89%, respectively. These rates are in the range reported in previous studies using different multimodal therapeutic approaches to LACC patients (3, 17, 18, 31, 32).

The retrospective nature represents a limit of the present paper, and has to be considered in toxicity evaluation. Another limitation of the study, moreover, is represented by the impossibility of performing a statistical analysis between the different stages of the disease, due to the limited number of patients: this could be a bias, considering the wide difference in prognosis of the different stages of disease.

No grade 3-4 acute GI and genitourinary toxicity were reported in our series, while only five patients (3.4%) developed grade 3 haematological toxicity that led to temporary treatment interruption. With the limits inherent in the risk of under-reporting, the rate of late toxicity appears quite encouraging comparing to standard treatment, with only 8.8% of genitourinary grade 3 complication. Indeed, several studies on exclusive CRT have reported severe late toxicity ranging from 10 to 18.3% with a predominant pattern of intestinal toxicity (13% grade 3–4 complications), and vaginal toxicity (20% grade 3–4 complications) (33–35).

The reduction of acute GI toxicity compared to literature data could be related to the introduction of intensity modulated techniques and the development of more efficient image-guided radiotherapy protocols (13).

Patients with pathological nodal involvement after CRT experienced statistically significant worse outcomes in terms of local control, metastasis-free survival, and overall survival. These results underscore the need to stratify patients into risk classes in order to pave treatment pathways tailored according to the predicted outcome. Recently, a radiomics model for LACC patients undergoing neoadjuvant CRT was developed analyzing T2-weighted 1.5 T MRI acquired before treatment start, appearing to be a reliable tool in pCR prediction after neoadjuvant CRT (36). Taken into consideration that our study showed a 46% pathological complete response, these response prediction models could play a fundamental role in the management of patients with LACC by identifying patients who could benefit from lower doses, resulting in less toxicity. Based on this assumption, new dose delivery and targeting paradigms could be proposed and better treatment outcomes may be achieved, as already demonstrated in rectal and cervical cancer (36–38).

## Conclusions

Neoadjuvant chemoradiation seems to be a promising strategy in the management of locally advanced cervical cancer, with a high rate of pathologically assessed complete response and a very encouraging local control rate with acceptable toxicity. The worst outcome in patients with persistent pathological nodal involvement after neoadjuvant CRT underscored the need for further investigations with the aim of improving outcomes in this subgroup of patients.

In this scenario, it must be acknowledged that the ongoing LARA 4.0 prospective multicentric phase II trial (Eudract number: 2020-002300-40; www.eudract.emea.europa.eu) is testing the efficacy tolerability of simultaneous integrated boost on primary and positive lymph nodes in neoadjuvant CRT for patients with locally advanced cervical cancer.

## Data Availability Statement

The raw data supporting the conclusions of this article will be made available by the authors, without undue reservation.

## Ethics Statement

The studies involving human participants were reviewed and approved by Comitato Etico dell’Università Cattolica del Sacro Cuore - Policlinico Universitario A. Gemelli - Roma. The patients/participants provided their written informed consent to participate in this study.

## Author Contributions

Conceptualization, AN and RA; methodology, LB; validation, GM and MAG; formal analysis, AN and RA; investigation, AN; data curation, SR, AN, NB, MC, and LT; writing—original draft preparation, AN; writing—review and editing, RA, GM, and MAG; visualization, GM, LB, and GF; supervision, VV and MAG; project administration, RA and AN. All authors have read and agreed to the published version of the manuscript.

## Conflict of Interest

The authors declare that the research was conducted in the absence of any commercial or financial relationships that could be construed as a potential conflict of interest.

## Publisher’s Note

All claims expressed in this article are solely those of the authors and do not necessarily represent those of their affiliated organizations, or those of the publisher, the editors and the reviewers. Any product that may be evaluated in this article, or claim that may be made by its manufacturer, is not guaranteed or endorsed by the publisher.
